# A randomized controlled trial examining consumers’ perceptions and opinions on using different versions of a FoodFlip© smartphone application for delivery of nutrition information

**DOI:** 10.1186/s12966-020-0923-1

**Published:** 2020-02-12

**Authors:** Mavra Ahmed, Angela Oh, Lana Vanderlee, Beatriz Franco-Arellano, Alyssa Schermel, Wendy Lou, Mary R. L’Abbé

**Affiliations:** 1 Department of Nutritional Sciences, Faculty of Medicine, University of Toronto, Medical Science Building, 1 King’s College Circle, Room 5368, Toronto, ON M5S 1A8 Canada; 2 School of Public Health and Health Systems, University of Waterloo, Waterloo, N2L 3G1 Canada; 3 Dalla Lana School of Public Health, University of Toronto, Toronto, M5T 3M2 Canada

**Keywords:** Front-of-pack labelling, Smartphone application, Mobile health, Interpretative nutrition rating system

## Abstract

**Background:**

Food labelling is a common intervention to improve diets, where the back-of-pack Nutrition Information Panel (or Nutrition Facts table (NFt)) provides comprehensive nutrition information on food packages. However, many consumers find it difficult and time-consuming to identify healthier foods using the NFt. As a result, different interpretative nutrition rating systems (INRS) may enable healthier food choices and it is essential that consumers have the tools to allow for easily accessible nutrition information. The objective of this study was to examine consumers’ perceptions of different (INRS) for delivery of nutrition information using different versions of a smartphone app, FoodFlip©.

**Methods:**

This study was part of a larger randomized controlled trial examining consumer perceptions of different INRS on food products. A nationally representative commercial sample of 2008 Canadians were randomized to one of four INRS intervention groups: 1) traffic light, 2) health star rating, 3) ‘high-in’ warning labels or 4) no INRS (NFt only; control) and asked to scan or enter 20 products into FoodFlip© from a list of food products provided to them with varying levels of healthfulness. After completing the app task, participants were asked a series of 7-point Likert-scale and open-ended questions to provide opinions on the usability and functionality of the app.

**Results:**

Of the survey sample of 1997 participants, 95% (*n* = 1907) completed the app task, with similar number of participants in each treatment group. The mean age was 40 ± 12 years with no differences in sociodemographic characteristics between treatment groups. The health star rating ranked significantly lower in comparison to the other treatment groups in terms of usefulness (OR, 95% CI -0.67, 0.52–0.85), believability (0.59, 0.46–0.75), and understanding (0.55, 0.44–0.71) (*p* < 0.001). The health star rating (1.20, 0.94–1.53) and control (NFt) (1,1,1) ranked significantly lower than the traffic light or the ‘high-in’ warning labels for their ability to compare the healthfulness of products (*p* < 0.001).

**Conclusion:**

This study demonstrated Canadian consumers’ preference for a nutrient-specific system (i.e. traffic light or ‘high-in’ warning labels). The app, which was liked by majority of the participants for its functionality and usability, has the potential to support healthy dietary decision making and may also encourage reformulation.

**Trial registration:**

NCT03290118 (Clinicaltrials.gov).

## Introduction

Food labelling is among the most common interventions used by governments to improve diets [1] and the mandatory back-of-pack nutrient declarations (i.e. Nutrition Facts table (NFt) in Canada; (also known as the Nutrition Information Panel, Nutrition Facts Panel) provides comprehensive nutrition information on the food packages [2], however, many consumers are unable to interpret the NFt to inform their dietary choices [3, 4]. Food package labels also contain multiple forms of nutrition information including mandatory ingredient lists and voluntary manufacturer marketing, which can further add to the confusion of interpreting the nutrition information found on food packages [5]. Numerous studies have shown the influence of consumer understanding and demographic characteristics on use of the NFt and other nutrition marketing on food labels (e.g. health claims, nutrient content claims and front-of-pack labels) [5–11]. These studies show that although consumers can make simple comparisons between similar products, many have difficulties understanding how to evaluate the information presented on the package with respect to healthfulness [8, 12–14]. Although, the majority of Canadians indicate that they use the information on the food product to make healthier choices, the complexity of nutrition information, in addition to the diverse forms of information on labels and time pressures often complicate the identification of healthier choices [3, 15].

In recent years, additional labelling initiatives that complement the mandatory back-of-pack nutrition labelling have been proposed to help consumers achieve more healthful diets [16, 17]. In particular, interpretative nutrition rating systems (INRS) on the front of food packages, commonly known as front-of-pack labels, has emerged as a promising intervention to influence consumer behaviour and product reformulation [18]. Three general types of INRS can be found on food packages: nutrient specific systems that show the amount per serving or per 100 g of select nutrients (e.g. multiple traffic light system or ‘high in’ warning labels (usually displayed when thresholds for amounts of saturated fat, sodium and/or sugar are exceeded); summary indicator systems that use a single symbol or score to provide information on the overall nutritional quality of the food or beverage product (e.g. health star rating); or hybrid systems that combine characteristics of the preceding systems (e.g. traffic light system with percentage of guideline daily amounts) [18]. INRS, such as single/multiple traffic lights, health star ratings or ‘high in’ warning labels have the potential to help consumers understand the nutrient data and compare the nutritional quality or healthfulness of food and beverage products in an easy-to-understand and accessible format [18, 19]. Recently, regulations requiring mandatory ‘high in’ warning labels have been proposed by Health Canada on the front of all food and beverage products that exceed established nutrient thresholds for saturated fat, sodium and/or sugar [20]. These INRS symbols or logos provide interpretive information regarding the healthfulness of food products and have been proposed to help overcome the known limitations of the NFt (e.g. difficulties in evaluating nutrient levels relative to dietary recommendations and making comparisons between products) [21]. The proposed regulations were published in Canada Gazette, Part I in 2018 [22], although they have not been implemented or finalized.

Canadians commonly seek out food and nutrition information from easy to access sources that include the web and print media [23]. Considering the recommendations for a single, standardized INRS front-of-pack system in the form of simplified ‘high-in’ warning label symbols [24, 25], mobile technology represents an innovative opportunity to enable healthier food selection by consumers without relying on the voluntary adoption of a uniform system by the food industry or the need for government regulations and can also be used during the transition period prior to implementation of government regulations, which would have taken 5 years to implement in Canada, once regulations had been finalized [22]. In Canada, there is an ever-increasing access to web and mobile technologies [26]. In 2018, 86% of Canadians owned a smartphone, with usage increasing significantly every year [26, 27] . Research shows that tablet and smartphone technologies (mobile apps) are feasible and accepted across all age and socioeconomic groups [28]. Consumer nutrition mobile apps are one means to help promote a healthy lifestyle across cultural, literacy and numeracy barriers [29]. Use of consumer nutrition mobile apps also has the potential to reduce healthcare costs and reach a wide spectrum of sociodemographic strata, including those who may be at a higher risk of less healthy dietary behaviours (e.g. consumption of unhealthy diet, lack of physical activity) [30].

Several studies have indicated positive health behaviour change (e.g. selection of healthier food choices) in response to usage of consumer nutrition mobile apps (health-related apps) [31, 32]. For example, the SaltSwitch smartphone app was shown to be effective in supporting individuals with cardiovascular disease in choosing lower-sodium foods [33]. Similarly, FoodSwitch, a smartphone app developed in Australia which displays nutrition information using different INRS resulted in a large number of downloads including a positive retention rate of its usage and has been shown to empower consumers to make healthier food choices [34]. Taken together, this evidence suggests that the use of consumer nutrition mobile apps may help enable healthy dietary choices at the point of purchase in a time-constrained environment (such as while grocery shopping) [35]. The success of FoodSwitch indicates that the public is interested in making healthier food choices during grocery shopping [34]. This app has also been launched in other countries such as China, Fiji, UK, India, New Zealand, South Africa, although results of its use are not yet available [36, 37]. In addition to enabling healthier food selection, data collected from FoodSwitch has shown the food industry to be lagging behind on their targets for food reformulation (e.g. sodium) [38] and therefore, consumer nutrition mobile apps also have the potential to drive food reformulation. Other forms of consumer nutrition mobile apps, including FoodSMART [39], MyNutriCart [40], SmartAPPetite [41], have all been shown to improve food selection or awareness and lead to positive behaviour change.

With the considerable potential for mobile apps to enable healthier dietary behaviours, helpful tools and easily accessible information via apps are needed to help Canadians make healthier food choices and thereby manage their diets. Although several Canadian computer and mobile-based tools and interventions have been developed to comprehensively evaluate nutritional status (e.g. Nutri-eSCREEN, EaTracker) [42–46], none of these are designed to act at the point of purchase to accelerate the comparison of the healthiness of products and support consumers in making timely healthier food choices. Moreover, there is a paucity of research assessing consumers’ perceptions on the use of smartphone apps to enable healthy food choices and in evaluating whether Canadians can use nutrition focused apps to interpret the complicated information presented on food labels. Additionally, in the absence of implementation of front-of-pack nutrition labels in Canada, and given the increased consumption of saturated fat, sodium and total sugar from processed foods [47], we developed the FoodFlip© nutrition mobile app to provide consumers with point-of purchase access and easy-to-understand nutrition information in the form of INRS. Therefore, the objective of the current study was to examine consumers’ opinions and perceptions on different INRS using a food information smartphone app, FoodFlip©, to provide and compare nutrition information of food products in the Canadian food supply.

## Methods

### FoodFlip© app design and functions

#### Identification of nutritional information on Canadian foods and beverages

Nutrition information on Canadian foods and beverages contained within the FoodFlip© app was collated from the University of Toronto’s Food Label Information Program (FLIP) 2013 database (*n* = 15,342, 48). FLIP 2013 contains nutrient content (from the NFt), ingredient list, universal product code, health/nutrient content claims, etc. among other information on Canadian pre-packaged foods and beverages for private-label and national brand foods. The database is described in detail elsewhere [48].

#### Categorization and search function of foods and beverages in the FoodFlip© app

The process of developing the FoodFlip© app involved categorizing all packaged foods within the FLIP database into product specific major categories (*n* = 19), sub categories (*n* = 101) and minor categories (*n* = 397) in order to allow consumers to easily locate products in consumer-friendly categories. For example, for beverages, the categorization of products was as follows: Beverages as major category, Hot Drinks or Soft Drinks as sub-categories and Coffee, Tea, Hot Cocoa or Iced Tea, Sugar-Sweetened Soft Drinks or Sugar-Free Soft Drinks as minor categories. The food categorization system was based on merging Health Canada’s Schedule M food categories [49], Canada’s sodium reformulation target categories [50], and more specific subsets of food categories (based on the iterative development process). Categories were modified if found to be ambiguous or difficult for participants to find during the beta-testing of the app (see below). Health Canada’s Schedule M food categories is a document that lists the reference amounts of foods typically eaten in one single setting for 153 categories of foods [49]. This document is primarily used as a criterion to determine the display of the nutrition information on the back-of-pack and as a criterion for nutrient content claims and health claims [49]. Canada’s sodium reformulation target categories lists the sodium benchmark targets for sodium focused food categories [50]. The FoodFlip© app allowed users to search for products using three different functions: 1) using a type-ahead function in the search field for the product name or manufacturer; 2) search using major, sub and minor categories; or 3) scan the product barcode using the smartphone camera.

#### Interpretative nutrition rating systems and healthfulness comparison function

**Fig. 1:**
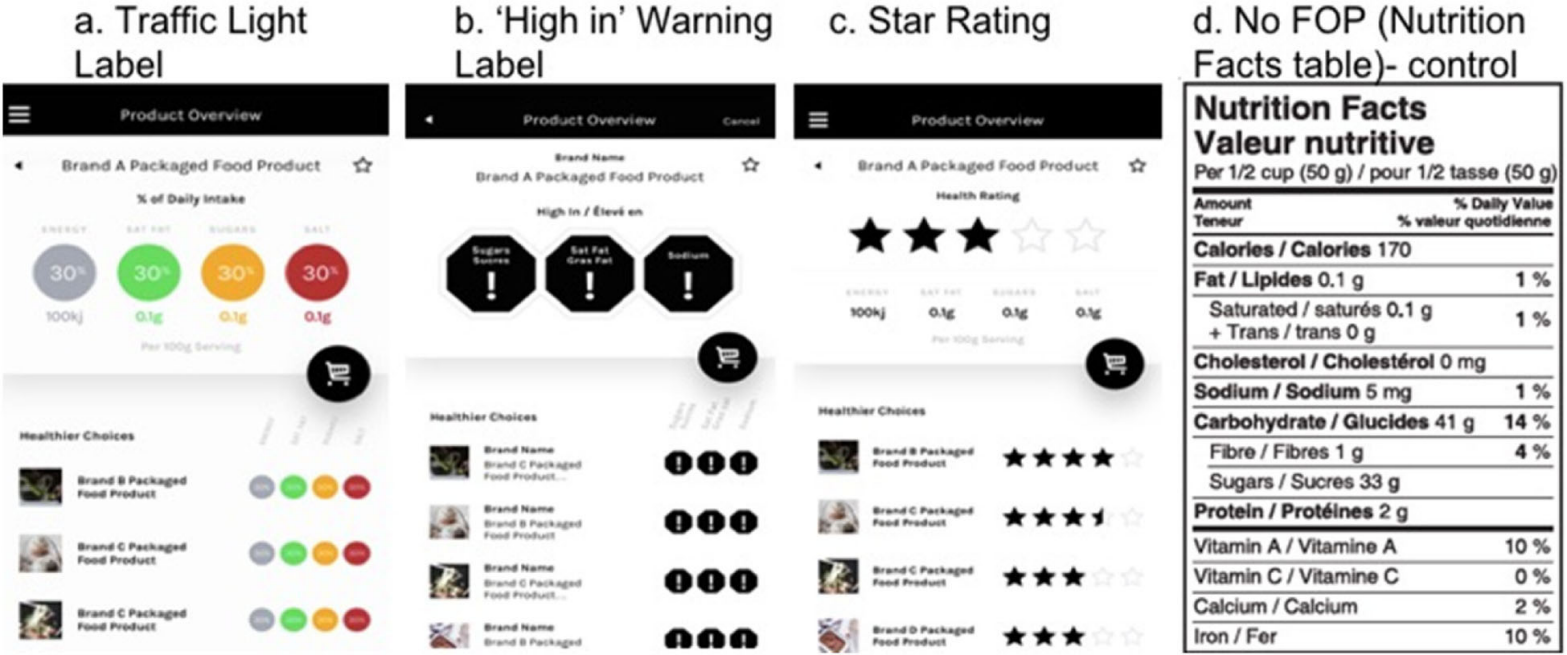
App screenshots of FoodFlip© for each of the interpretative nutrition rating system (INRS): **a** Traffic Light Label, **b** ‘High in’ Warning Label, **c** Star Rating and **d** No Front-of-Pack (Nutrition Facts table (NFt) (Control; without healthfulness comparison feature))

Although many different formats of INRS have been identified worldwide; traffic light labels, health star rating or star rating (adapted from Australia/NZ health star rating) and ‘high in’ warning labels (Fig. 1) were selected for the current study, given that: 1) traffic light, star rating and ‘high in’ warning labels reflect different FOP symbols currently in use around the world [51, 52]; 2) regulations requiring ‘high in’ warning labels were recently proposed by Health Canada as part of Canada’s Healthy Eating Strategy [20]; 3) supportive evidence for all three systems suggests potential acceptance or usage by consumers [19, 52] and; 4) all three systems could be formatted to be congruent with Canadian food standards and regulations. The app displaying the Canadian NFt was provided as a control. All products in the FLIP database were assessed according to the criteria set by the respective organization to assign the traffic light [53], star rating [54, 55] or Canada’s ‘high-in’ warning label rating [25].

The healthfulness comparison feature (a feature that allows consumers to look at other comparable products’ nutritional information displayed below the nutrition information for the selected product) was designed to enhance consumers’ decision quality with minimal investment of time or effort, helping them to choose products of higher nutritional quality quickly and efficiently. In the FoodFlip© smartphone app, the ‘searched for’ product as well as the comparable products’ nutritional information was displayed using one of the following INRS systems (Fig. 1).

### Beta-testing of the FoodFlip© app

The FoodFlip© project was launched with the goal of developing a mobile nutrition app as a technological solution for quick and easy display of nutrition rating systems with the healthfulness comparison feature to help consumers select healthier products. The key considerations employed in developing the app were the method of user interaction with the mobile app, the platforms the app would be developed for and the nutritional algorithms designed to categorize, and score packaged foods within the FLIP database for incorporation into the app.

Initial app development and programming for the health stars and the traffic light INRS was completed in August 2016 and was designed to be compatible with both Android and iPhone operating platforms. FoodFlip© was updated to include the ‘high in’ warning labels in March 2017, after the release of the Healthy Eating Strategy by Health Canada [20] in order to test the effectiveness of this type of system on consumer understanding of product healthfulness and inform discussions on the Canadian policy.

An initial test version of the mobile phone app was deployed for user acceptability testing (in September and October 2016) by a convenience sample of individuals (friends and family members of the research team). Participants were asked to download the app (link provided by email) and scan pantry or grocery store shelves using the app. Twenty-three participants expressed interest in downloading the app, and 18 completed the task and answered open-ended questions on usability of the app.

From this beta-testing of the app, 67% of participants liked the app, and 50% said they would use the app. Majority (83%) said that product categories were ‘easy to understand’ but 50% of the traffic light group indicated preference for ‘one’ symbol encompassing all nutrients. Participants also commented that it was difficult to find the product in the app. These initial results identified several challenges with deploying the app on a larger scale, particularly the need for updated information on product SKUs and the product names, as many of these had changed since the most recent data input in 2013. Testing also revealed that consumers would benefit from a ‘type ahead input field (autocomplete)’ feature in the search function of the app; these modifications were incorporated in the final FoodFlip© app used in the study.

### Study design

The information and data presented (i.e., the study described in this specific manuscript) was part of a larger randomized controlled trial using an online survey panel to examine consumer perceptions of interpretative nutrition rating systems (INRS) on the front of food packages. Briefly, in the larger randomized controlled trial, a randomized parallel-group design was used in which participants were randomized to one of four nutrition labelling conditions (same as for the app task described below): 1) traffic light, 2) health star rating, 3) ‘high-in’ symbol or 4) no INRS (Nutrition Facts table (NFt)) (control). In the larger randomized controlled trial, participants were given a task to complete using the smartphone app as a means to educate them on the use of the various labelling systems, and then were asked to complete two tasks using an online survey questionnaire, which asked questions about consumer perceptions and intention to purchase various food products. A detailed description of the study can be found on clinicaltrials.gov (NCT03290118). Prior to recruitment, the study was approved by University of Toronto Research Ethics Board (Approval # 34393).

In the study, a nationally representative commercial sample of 2008 Canadians were randomized to one of four INRS intervention groups: 1) traffic light, 2) health star rating, 3) ‘high-in’ symbol or 4) no INRS (Nutrition Facts table (NFt)) (control). Participants remained in the same intervention groups for all the survey questions. Randomization was conducted by Leger Marketing, a commercial sampling firm, using an online computerized system in a 1:1:1:1 ratio.

Baseline data collection comprised of self-reported socio-demographic variables (including gender, age, ethnicity, self-reported height/weight to calculate body mass index (BMI), education, health literacy measured using the Newest Vital Sign© (NVS) questionnaire, income, province, language primarily spoken at home, living with children). Newest Vital Sign© is a six-questions health literacy questionnaire based on the NFt and has been validated for use in Canada [56]. Based on NVS score (a maximum score of six), participants were categorized as follows: a) low health literacy (score 0–1), b) “possible” low health literacy (score 2–3), or c) “adequate” health literacy (score 4–6) [56]. The Newest Vital Sign© was adapted for online use and no audio was provided for questions. Participants completed and answered survey questions for several different tasks, however, this study presents the results for the task limited to the use of the smartphone app, FoodFlip©.

### Study participants

Participants were recruited by Leger Marketing (a professional recruitment firm) from their Leger Web Panel of approximately 400,000 Canadian adults and directed to the study website. All study communication took place through Leger Marketing. Participants were eligible for the study if they were 18 years of age or older, spoke English as their primary language, resided in Canada (excluding Northern territories), provided informed consent, did some of the grocery shopping in the household (defined as at least twice a month), owned a smartphone (version iPhone 3 or later or Android) and were able to complete the survey (consent, socio-demographic information, experimental tasks, and app-related questions) on a minimum screen size of 9.7 in.. A nationally representative sample based on 2011 census data for age, sex and region and who had access to smartphone and met the eligibility criteria for the study were recruited. Recruitment was completed between September 2017 and October 2017. Participants received $10 or the equivalent in Air Miles® from Leger Marketing when the survey was completed. Participation was voluntary and participants could withdraw at any time. Participants were assigned a study ID to permit linkage of data for all the survey questions.

The planned sample size was 2000 randomized individuals assigned in a 1:1:1:1 ratio to one of the four conditions (traffic light label, health star rating, ‘high in’ warning label or no INRS label - all intervention groups had access to the NFt by clicking an icon) designed and powered for the larger RCT. The sample size of 2000 participants, with 500 participants in each of the four INRS treatment groups for the smartphone app, was estimated with 90% power to detect a 0.4 unit difference in perceived healthfulness between the INRS labelling conditions (s.d. =1.5). Our sample size of approximately 500 participants in each of the treatment groups was more than sufficient to detect differences in consumer perceptions.

### App task

Participants were directed to download the FoodFlip© smartphone app and randomized to one of the four intervention groups (traffic light, health star rating, ‘high-in’ warning label or NFt) after providing informed consent. All participants provided information on socio-demographic characteristics immediately before completing the app task and completed the Newest Vital Sign© health literacy questionnaire immediately after the app task.

#### App task

Participants were asked to scan or enter the 20 products into FoodFlip© from a list of food products provided to them with varying levels of healthfulness, based on Food Standards of Australia and New Zealand nutrient profiling model criteria [54] (Table 1). The Food Standards of Australia and New Zealand nutrient profiling model criteria was used as this has been previously validated [57] and takes into account both nutrients-to-limit as well as positive nutrients [54].

**Table 1:** List of 20 food product types with varying levels of healthfulness (based on Food Standards of Australia and New Zealand nutrient profiling model criteria), that participants were asked to enter or scan into the smartphone app

a. Jelly Sponge Cakes	
b. Lemon Iced Tea	
c. White Bread	
d. Ranch Dressing	
e. Frosted Flakes	
f. All Dressed Potato Chips	
g. Crunchy Granola Bars	
h. Hash Browns	
i. Mixed Vegetables	
j. Hot Dog Buns	
k. Hummus Chickpea Spread	
l. Peaches & Cream Corn	
m. Greek Yogurt	
n. Red Kidney Beans	
o. Popcorn	
p. Chocolate Chip Cookies	
q. Thin Crust Pizza	
r. Chocolate Ice Cream	
s. French Vanilla Stirred Yogurt	
t. Stix Cheese Flavour	

There was no time limit set for the completion of this task. As this task was to be completed before participants could continue with the rest of the survey as part of the larger study, it is likely that participants completed the app task at home. However, considering that once the app was downloaded, participants had unlimited access to the app, it is possible that they conducted this task in other locations, such as by scanning products at the grocery store. Data on the location where a product was scanned or searched were not collected as part of this study. After completing the app task, participants were asked a series of 7-point Likert-scale questions on app use in relation to the specific INRS (one of the four intervention groups). The primary outcomes were the 7-point Likert-scale responses to the usability and functionality statements on app use (*n* = 8 statements). Self-reported opinions and challenges, using open-ended questions, about the app were also collected. A ‘don’t know/ I prefer not to answer/refused’ option was provided for all questions.

Participants were asked to rate their agreement with eight questions about the app using a 7-point Likert Scale (1 = completely disagree, 7 = completely agree). App quality was assessed using two user-interactive measures of 1) usability and 2) functionality from the multi-dimensional framework for assessing health app quality from Grundy et al., 2016 [58]. Usability was defined as the ‘quality of user interface’ which assesses the user satisfaction and user engagement with the app. The following statements were rated in the usability features of the app: the product search feature was easy to use, the barcode scanner feature was easy to use (if you used this feature), the app was easy to use, and the app was confusing. Functionality was defined as the operability of the app according to its purpose or design. In this study, functionality measures the user-evaluated reliability of the nutritional information and comparisons of products (i.e. the ability to rate the perceived healthfulness of foods). The functionality features of the app were assessed using agreement with the following statements: the app provided me with information I can use, the app was believable, the app helped me to understand the nutrient levels, and the app helped me to compare the healthiness between similar products.

### Statistical analysis

Data were tested for normality and descriptive statistics were calculated for demographic characteristics of participants (in each of INRS intervention group) and for their responses to the 7-point Likert scale for the pre-defined set of app statements. Socio-demographic variables of age, gender, ethnicity, body mass index (BMI), education, income and health literacy were identified a priori as associated with the use of mobile technologies and were included as covariates [28, 59]. There were no differences in province, language and number of dependent children between the four INRS groups (data not shown). BMI was calculated from self-reported height and weight data provided by the participants.

Baseline characteristics are presented as means with standard deviations (SD) for continuous variables and as percentages for categorical variables. Differences in age by INRS intervention groups were analyzed using the Kruskal-Wallis test. Differences in sociodemographic characteristics (health literacy score, gender, ethnicity, BMI, education, and income) of participants by INRS intervention group were analyzed using a chi-square test. The category of ‘other identity’ in gender was not considered for the overall analysis since there was only one participant per intervention group who self-identified in the category.

#### Consumer perceptions of app usability and functionality

Ordinal Logistic Regression (with Bonferroni adjustment for multiple comparisons, *α* 0.05/8 tests per intervention group = 0.006) was used to assess for associations between the INRS systems with the 7-point Likert scale responses, controlling for the following covariates: gender, ethnicity, BMI, education, income, age, and health literacy score. The 7-point Likert scale responses of the pre-defined set of app-related statements (*n* = 8) were treated as ordinal dependent variables whereas the INRS systems were treated as the categorical independent variables. Multicollinearity was not found between the covariates or independent variable therefore, all the covariates were included in the final model.

#### Assessment of app usability and functionality

Cronbach’s alpha was used to assess the reliability of the pre-defined set of app-related statements (for usability and functionality). The Likert scale ratings of 5, 6, and 7 were added to compute the proportion of participants who overall ‘agreed’ with the pre-defined set of app statements. Usability and functionality by self-reported challenges/opinions, patterns or trends were also identified in the open-ended responses of participants by thematic analysis. Chi-square tests were used to test usability and functionality with gender, age, education and income level (key factors identified in influencing the use of technology in decision making [59]).

All data were analysed using SPSS Statistics (version 24, 2016; IBM Corporation®, Armonk: NY, USA). Considering the multiple pairwise comparisons of the intervention groups and large sample size, a *p*-value of < 0.01 was considered significant.

## Results

### Study participants

**Fig. 2:**
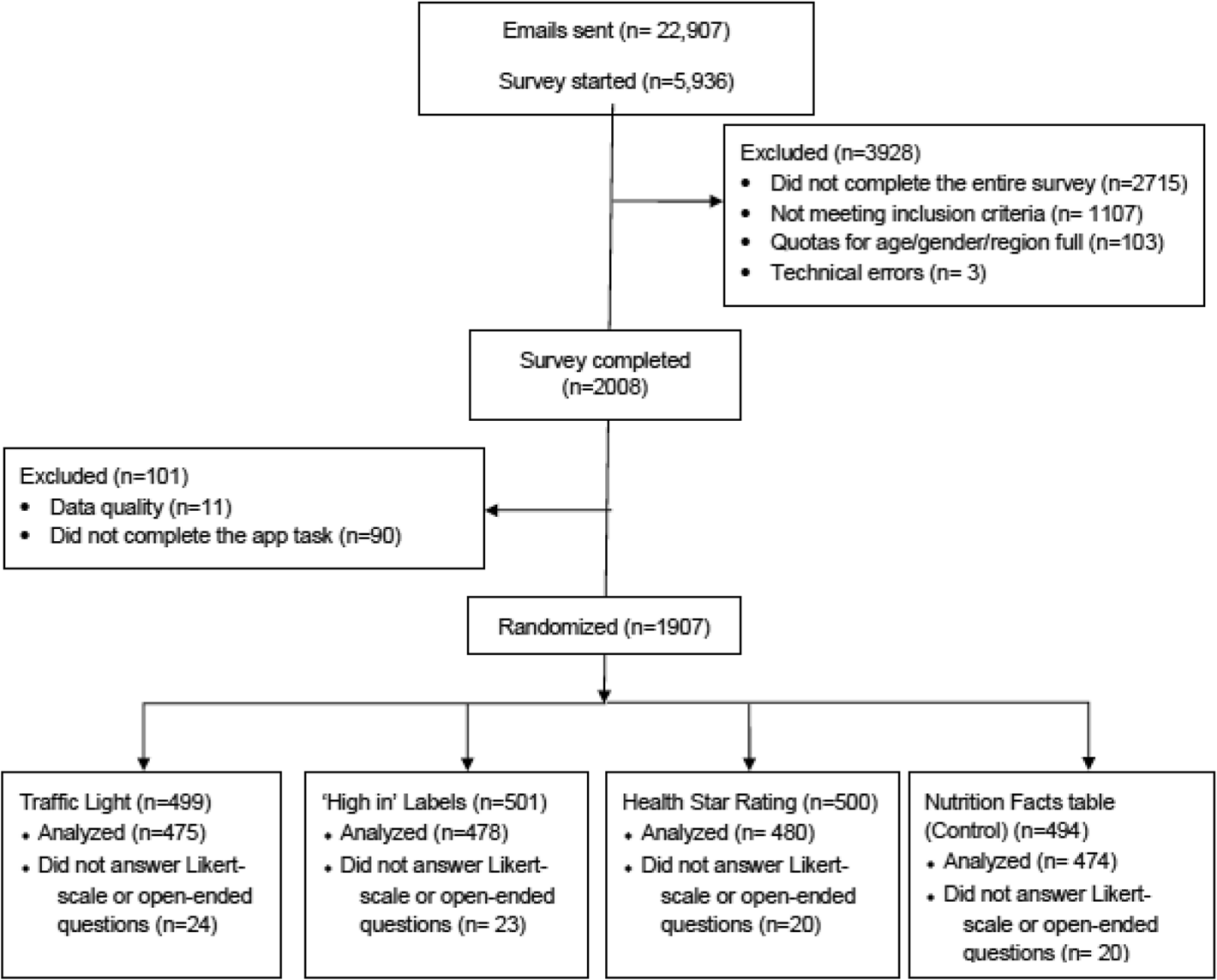
CONSORT diagram

Of 22,907 email invites sent out by Leger Marketing, 5936 started the link, from which 3928 were excluded as follows: 2715 did not complete the entire survey, 1107 were screened out using eligibility criteria, 103 were screened out because the quotas for their age/gender/region were full, and 3 had technical errors, for a total of 2008 participants who completed the entire survey (Fig. 2).

Of the 2008 participants who completed the survey, 11 participants were removed from the sample for data quality. Criteria to exclude data included; 1) if the participant refused to complete 2 or more of the three main experimental tasks; or 2) if the participant responded ‘Don’t know’ to the three main experimental tasks in the survey and also responded ‘Don’t know’ to 3 or more of 5 questions considered by the research team to be variables that are not typically sensitive for participants to report (Fig. 2).

From the survey sample of 1997 participants, 95% (*n* = 1907) completed the app task, with similar number of participants in each intervention group (Table 2). The mean age was 40 ± 12 years and the baseline characteristics (gender, ethnicity, BMI, education, the health literacy score as measured by the Newest Vital Sign© and income) were similar between the participants randomized to the four intervention groups (Table 2). The median time spent using the app to search for 20 food products was 12.5 min.

**Table 2:** Characteristics of Participants by App Intervention Group (Traffic Light Label, ‘High in’ Warning Label, Star Rating and Control (NFt))

Characteristics	Traffic Light (*n* = 475)	High in Warning (*n* = 478)	Star Rating (*n* = 480)	Control (NFt) (*n* = 474)	*p*-value
Number of participants who used the app in each condition	475 (95%)	478 (95%)	480 (96%)	474 (96%)	
Age (years) (mean (SD))	39 ± 13	39 ± 12	40 ± 13	40 ± 12	0.71
Gender					
Male	213 (45%)	220 (46%)	225 (47%)	242 (51%)	0.52
Female	261 (55%)	257 (54%)	255 (53%)	231 (49%)	
Ethnicity					
White	342 (72%)	329 (69%)	316 (66%)	334 (70%)	0.07
Other	132 (28%)	141 (29%)	153 (32%)	130 (27%)	
Not stated	1 (0.2%)	8 (2%)	11 (2%)	10 (2%)	
Calculated BMI^1^					
< 18.5	13 (3%)	10 (2%)	25 (5%)	10 (2%)	0.18
18.5 to 24.9	185 (42%)	185 (42%)	190 (42%)	175 (39%)	
25 to 29.9	123 (28%)	133 (30%)	134 (30%)	147 (33%)	
> 29.9	123 (28%)	120 (27%)	100 (22%)	115 (26%)	
Not stated	31 (6%)	30 (6%)	31 (6%)	29 (6%)	
Education					
Did not graduate high school	12 (2.5%)	5 (1%)	7 (1.5%)	11 (2%)	0.54
High school graduate certificate/equivalent	79 (17%)	75 (16%)	87 (18%)	67 (14%)	
Trades certificate/diploma	22 (4%)	30 (6%)	20 (4%)	24 (5%)	
Community/technical college or CEGEP	123 (26%)	124 (26%)	127 (27%)	122 (26%)	
University (undergraduate degree)	168 (35%)	189 (40%)	184 (38%)	180 (38%)	
Post-graduate degree (Master, PhD)	70 (15%)	54 (11%)	54 (11%)	68 (14%)	
Newest Vital Sign Score (max 6)					
Low (0–1)	49 (10%)	53 (11%)	42 (8%)	48 (10%)	0.13
Likely Low (2, 3)	58 (12%)	46 (10%)	73 (15%)	72 (15%)	
Adequate (4–6)	367 (77%)	378 (79%)	364 (76%)	354 (75%)	
Income					
Under $49,999	129 (27%)	127 (27%)	122 (25%)	131 (28%)	0.04
$50,000 to $99,999	177 (37%)	180 (38%)	206 (43%)	154 (32%)	
Over $100,000	132 (28%)	129 (27%)	120 (25%)	161 (34%)	
Don’t know	6 (1%)	13 (3%)	6 (1%)	3 (0.6%)	
Refused	31 (7%)	29 (6%)	26 (5%)	25 (5%)	

Data presented as *n* (%) unless otherwise indicated. There were no significant differences between intervention groups for age, gender, ethnicity, BMI, education, health literacy score (measured by newest vital sign) and income, where *p* < 0.01 was considered statistically significant. Body Mass Index^1^ (BMI) calculated from self-reported height and weight data provided by the participants where underweight (< 18.5), normal weight (18.5 to 24.9), overweight (25 to 29.9) and obese (> 29.9)

### Consumers’ perceptions on use of the FoodFlip© smartphone application using different interpretative nutrition rating systems (INRS)

The health star rating intervention ranked significantly lower in comparison to the other intervention groups (‘high in’ warning label, traffic light label and control (NFt)) for usefulness of information, believability of the app, and understanding of the nutrient levels (*p* < 0.01) (Table 3). ‘High in’ warning and traffic light INRS interventions ranked significantly higher in comparison with both the star rating and control (NFt) for the ability to compare the healthfulness of products (*p* < 0.001). The INRS intervention groups did not differ in the ratings for the following statements: the product search feature was easy to use, I liked the barcode scanner feature, I found the app easy to use and I found the app confusing (*p* > 0.01) (Table 3).

**Table 3:** Consumers’ Perceptions of the FoodFlip© App Usability and Functionality

7-point Likert-scale statements	*X*^*2*^	exp (B)	95% Confidence Interval		*p*-value
			Lower	Upper	
Usability^1^					
The product search feature was easy to use	0.58				0.90
I liked the barcode scanner feature	2.74				0.43
Overall, I found the app easy to use	4.17				0.24
I found the app confusing	2.25				0.52
Functionality^2^					
The app provided me with information that I can use	18.12				0.00
Traffic Light		1.06	0.83	1.35	0.65
‘High in’ Warning		1.02	0.80	1.30	0.89
Star Ratings		0.67	0.52	0.85	0.00
Control (NFt)		1.00	.	.	.
I found the app to be believable	21.51				0.00
Traffic Light		0.90	0.71	1.16	0.42
‘High in’ Warning		0.93	0.73	1.19	0.56
Star Ratings		0.59	0.46	0.75	0.00
Control (NFt)		1.00	.	.	.
Using the app helped me understand the nutrient levels in the food	34.75				0.00
Traffic Light		1.07	0.84	1.37	0.58
‘High in’ Warning		0.78	0.61	1.00	0.05
Star Ratings		0.55	0.44	0.71	0.00
Control (NFt)		1.00	.	.	.
Using the app helped me to compare the healthiness between similar products	19.78				0.00
Traffic Light		1.67	1.30	2.13	0.00
‘High in’ Warning		1.49	1.17	1.91	0.00
Star Ratings		1.20	0.94	1.53	0.14
Control (NFt)		1.00	.	.	.

Data analyzed using Ordinal Logistic Regression to assess for associations of the INRS systems with the 7-point Likert scale responses, controlling for the following covariates: gender, ethnicity, BMI, education, income, age, health literacy score. The 7-point Likert scale responses (1 = completely disagree, 7 = completely agree) of the pre-defined set of app-related statements (*n* = 8) were treated as ordinal dependent variables whereas the INRS system was treated as the categorical independent variables. ^1^Usability was defined as the ‘quality of user interface’ which assesses the user satisfaction and user engagement with the app and ^2^Functionality was defined as the operability of the app according to its purpose or design for the study’s objective, measuring the user evaluation of comparing the nutritional information of products. *p* < 0.01 was considered statistically significant

### Usability and functionality of the smartphone application

**Fig. 3:**
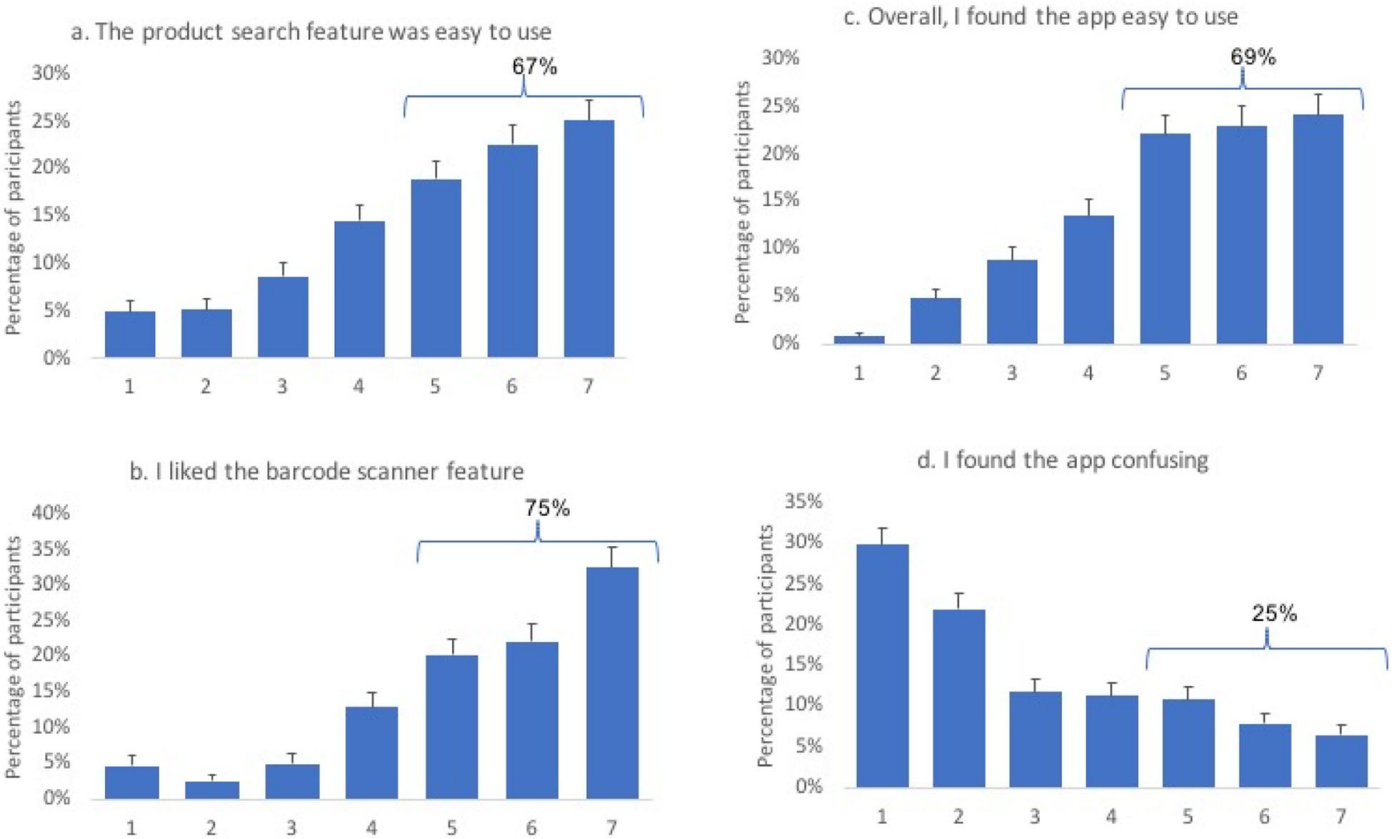
7-point Likert scale responses on the usability of the FoodFlip© smartphone application. Usability was defined as the ‘quality of user interface’ which assesses the user satisfaction and user engagement with the app. Four statements were used in assessing the usability features of the app: **a** ‘the product search feature was easy to use’, **b** ‘I liked the barcode scanner feature (if you used this feature)’, **c** ‘the app was easy to use’ and **d** ‘the app was confusing’. 7-point Likert scale ratings corresponded to completely disagreed (1) to completely agree (7)

With regards to the usability features of the app, 67% said that the product search feature was easy to use (25, 23 and 19% for the ratings of 7, 6 and 5, respectively). The majority of the respondents (75%) liked the barcode scanner function, corresponding to 33,22 and 20% ratings of 7, 6, and 5, respectively. Overall, 69% of the participants found the app easy to use (24, 23 and 22% for the ratings of 7, 6 and 5, respectively) and only 25% found the app to be confusing (6.5, 7.9 and 11% for the ratings of 7, 6 and 5, respectively) (Fig. 3).

**Fig. 4:**
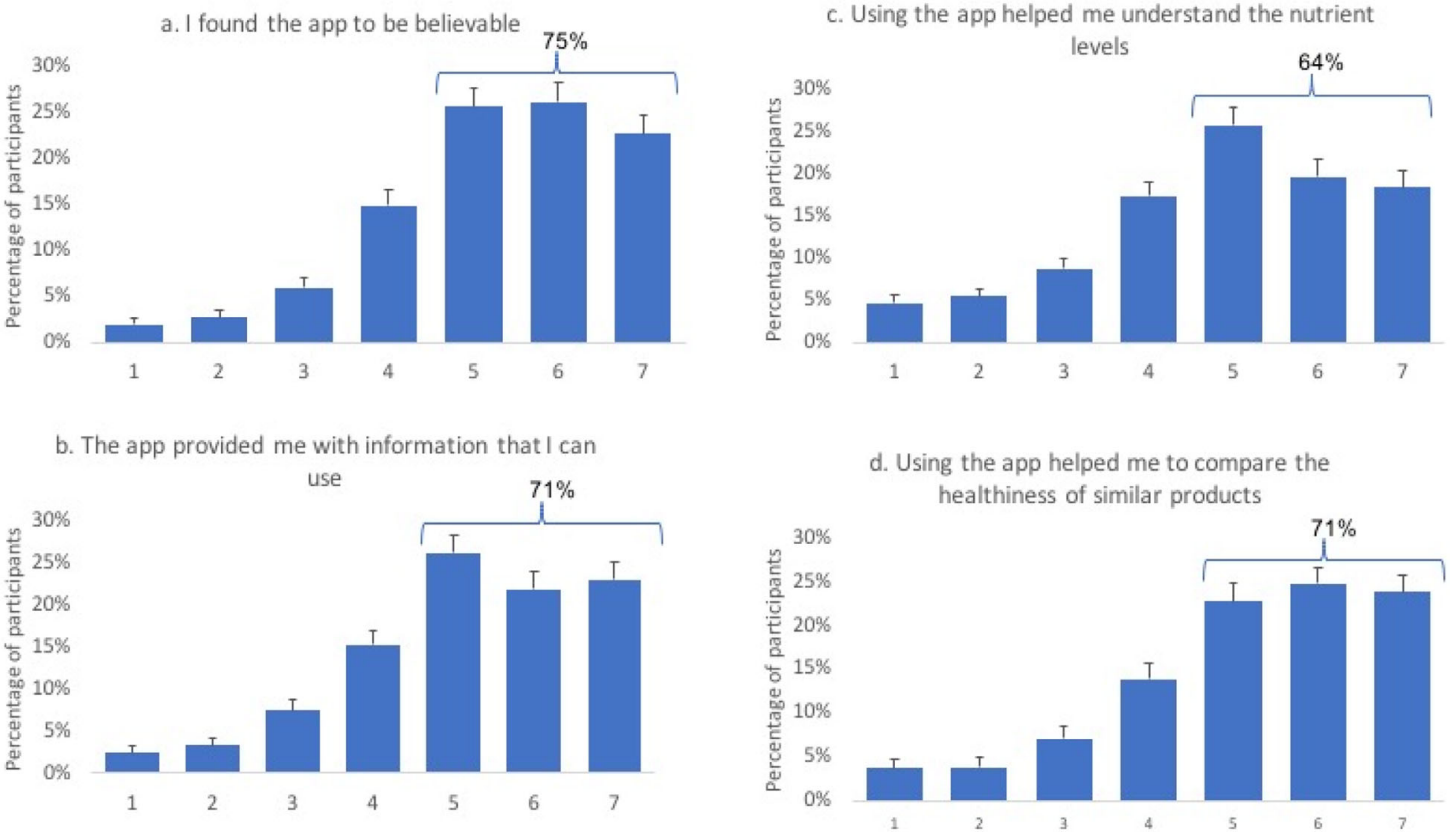
7-point Likert scale responses on the functionality of the FoodFlip© smartphone application. Functionality was defined as the operability of app according to its purpose or design and in this study, measures the user-evaluated reliability of the nutritional information and comparisons of products. Four statements were used in assessing the functionality of the app: **a** ‘the app provided me with information I can use’, **b** ‘the app was believable’, **c** ‘the app helped me in understand the nutrient levels’, and **d** ‘the app helped me compare the healthiness between similar products’. 7-point Likert scale ratings corresponded to completely disagreed (1) to completely agree (7)

For the functionality features, 71% indicated that the app provided them with information they can use (23, 22 and 26% for the ratings of 7, 6 and 5, respectively) and 75% found the app to be believable (corresponding to 23, 26, 26% for the ratings of 7,6 and 5). 64% said that the app helped them understand the nutrient levels (18, 20 and 26% rated this statement as 7, 6 and 5, respectively) and 71% indicated that the app helped them compare the healthiness of the similar products (24, 25, and 23% rated this statement as 7, 6 and 5, respectively) (Fig. 4). The 7-point Likert scale, measuring app usability and functionality on the pre-defined set of app statements, received a high Cronbach alpha (0.83).

**Fig. 5:**
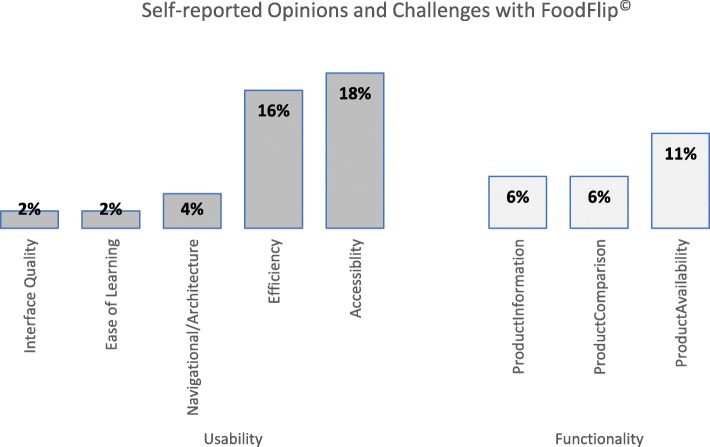
Self-reported opinions and challenges using the FoodFlip© app (*n* = 1438^1^). Bars show the proportion of participants who provided written responses to the question: “What are some of the challenges you had when using the app?”. Data grouped by thematic analysis and analysed using chi-square test for proportions/counts and presented as % (number of participants)

Participants were asked to comment on any additional features of the app in an open-ended format (self-reported challenges and opinions which were not tested in the pre-defined set of app statements), where 36% of respondents identified additional usability challenges as follows: 20% of participants found the search bar menu option the most challenging usability issue. Overall, 21% of respondents identified additional functionality challenges (not tested in the pre-defined set of app statements), where the major issue was the difficulty in finding the product in the app (13% of the total respondents) (Fig. 5). Gender, age, income or education did not differ in either of the self-reported usability or functionality measures of the app (*p* > 0.05). Although the different app types were rated similarly across most of the challenges, participants commented that the stars app required better nutritional info, and the control (NFt) app group found it did not allow for comparison of products.

## Discussion

The purpose of this study was to examine consumers’ perceptions of interpretative nutrition rating systems (INRS) on their ability to provide nutrition information when using the food information smartphone app, FoodFlip©. Overall, the results indicated that Canadian consumers preferred the display of traffic light or ‘high in’ warning nutrition specific INRS in the food information smartphone app for comparing the healthfulness of similar products, for understanding nutrient levels in foods and for providing useful nutritional information compared with the star rating. Furthermore, the app was accepted for its usability and functionality by majority (%) of the participants.

Health Canada recently released a proposal to mandate the ‘high in’ warning labels on the front of all food and beverage products exceeding nutrient thresholds [20] and although, there is promising evidence of the impact of the proposed ‘high in’ warning labels from other countries [52, 60, 61], data in Canada are limited. The results of this study demonstrated that participants who used a smartphone app that communicated nutrition information using traffic light or ‘high in’ warning nutrient specific INRS reported it to be more useful with respect to comparing the healthfulness of similar products than those who saw apps with the star rating and control (NFt) systems. The traffic light and ‘high in’ warning INRS also ranked significantly higher for providing nutritional information that participants can use and for understanding the nutrient levels in comparison to that of the star rating app but did not differ with the ranking of the control (NFt). These findings are similar to those of previous research conducted in USA, UK, or Chile, where consumers indicated higher preference for nutrient specific systems, such as traffic light labels or ‘high in’ warning labels in comparison to a summary INRS system or control [52, 62–70]. For example, a web-based survey simulating an online grocery store, administered to 1182 people in Chile, indicated improved participants’ ability to perform a healthful food purchase when randomized to FOP nutrition information (modified traffic light system or the Chilean warning system) in comparison to the control condition [52]. Research from USA have shown multiple traffic lights to perform better in comparison to other INRS (including a graded system, star ranking and a nutrition facts table-based approach) in helping consumers to identify the healthier products as well as understanding of the nutrient levels [71]. Data from the UK also indicated that the majority of consumers used multiple traffic lights to ‘avoid red lights’ similar to a high in warning FOP system [70]. Results from the present work are similar to other studies which did not find differences in consumer perceptions between the traffic light and ‘high in’ warning labels [19, 52], likely because both are nutrient specific systems that communicate information about key nutrients (e.g. sodium, saturated fat and sugar). Although investigations on comparative efficacy of traffic light and ‘high in’ warning labels are limited, some studies have shown that ‘high in’ warning labels, in comparison to traffic light labels, reduced the time in decision making of key nutrients by consumers and were more efficient in helping consumers identify less healthful products [72].

Findings from the present study did not indicate superiority of INRS as more useful or easier to understand in comparison with the control (NFt) when using the smartphone app. These results are in contrast to previous research from other countries which indicated the various types of INRS as more useful, easier to understand, and have the potential to lead to improvements in nutritional knowledge or in the nutritional composition of the purchased products in comparison to the nutrition information panel or table [19, 51, 52]. The high ratings for the control (NFt) app with respect to the useful nutritional information and understanding of nutrient levels is similar to the results of previous research from our group where NFt was found to be more consumer friendly with respect to helpfulness, credibility, liking and influence on purchase decisions [15]. This is likely a result of widespread access, familiarity and use of the mandatory NFt label in Canada; 42% of Canadians reported getting information on food and nutrition using the NFt, and 83% believe that the NFt is a credible source of nutrition information [23]. Similarly, recent findings from Statistics Canada indicated that 56% of Canadians consult the NFt [73], although many have difficulties understanding how to evaluate the nutrition information presented on the NFt [8, 14]. This reiterates that while consumers may report that information is useful, the extent to which they are able to synthesize and interpret that information does not always align with self-reported usefulness and is an important context for the current findings. Additionally, our findings relating to comparisons, usefulness and understanding should be considered in light of the sociodemographic characteristics of our sample, which were predominantly white and relatively highly educated. As a result, despite our finding that NFt did not differ from traffic light or ‘high in’ warning labels in usefulness and understanding of the nutrient levels, further analysis of our data indicated less use of NFt and nutrition information in those with lower NVS score in the companion study [74], suggesting that certain segments of the population may benefit from a more simplified presentation of nutrition information.

Examination of front-of-pack nutrition labelling schemes using a smartphone app are limited [75], as majority of the studies have utilized a web-based survey or used non-randomized designs to investigate the perception of front-of-pack labels. To our knowledge, only two studies have used a smartphone app to assess the effects of INRS on consumers’ real-time food purchases and to evaluate preference and utility of different INRS systems [19, 51]. Our results are in alignment with the findings related to participants’ perceptions from these studies, which found that the participants were more likely to find the INRS useful and easy to understand and that their nutrition knowledge improved as a result of using these INRS in comparison to the control (NFt), when using a smartphone app as the INRS delivery method. These authors, however, also found that neither traffic light nor health star ratings had a significant effect on the healthiness of consumer food purchases in a real-world setting compared to the control (NFt) [19, 51], although one study using smartphone apps to evaluate front-of-pack labels found that warning labels resulted in healthier food purchases, while the health star rating was perceived by participants as easier to understand in comparison to traffic light or daily intake guides [19].

Research has shown the potential of INRS to help consumers make healthier food choices although evidence on the preference for type of INRS have been mixed [52, 76]. For example, a web-based cohort of French participants rated Nutri-score, which is a color-coded summary INRS, as most favourable, followed by multiple traffic light (nutrient specific) and SENS (*Système d’Etiquetage Nutritionnel Simplifié*) (summary, graded and color coded symbols) [76]. Another internet-based survey administered to consumers in USA randomized to six intervention conditions (no condition, single traffic light, multiple traffic light, Facts up Front, NuVal and 0–3 star ranking) to better understand the influence of front-of-pack labels on consumers’ perceptions, found that both NuVal (graded) and multiple traffic light labels led to an increased accuracy in identifying the healthier of the two products, whereas multiple traffic lights also allowed a better understanding of nutrient levels [71]. Research regarding consumers’ perceptions of INRS or the use of front-of-pack nutrition labelling systems to enable healthy food choices have been mixed [51, 71, 76], suggesting that the preference for a specific INRS is likely dependent on population characteristics, such as food preferences, food purchases, availability of product type, nutrition knowledge, social marketing campaigns and education/awareness of nutrition campaigns. Several studies have also shown that the results of preference between different front-of-pack systems vary by country and ethnic groups [63–65]. This reiterates the importance of better understanding the user experience and tailoring the usability features of the smartphone app to the specific population.

Considering the usage of web and mobile technologies is increasing significantly every year in Canada, smartphone-based nutrition information applications have the potential to facilitate healthier food decision-making by Canadians. Several studies have shown an increased opportunity of smartphone applications for use in accessing nutrition information and diet monitoring (i.e. consumption of foods and beverages such as a mobile food record) [31, 46, 77]. Findings from this study demonstrated that the majority of participants accepted the app for its usability and functionality, attributes that allow for simple access to nutrition information enabling consumers to make healthier food choices with minimal investment of effort in real-time. In this study, gender, age, income and education level did not influence the use of app, indicating the potential of FoodFlip© for reaching a wide spectrum of sociodemographic strata. Research has also shown that consumers with lower level of education or socio-economic status tend to benefit from nutrition information apps [78], although they may also be less likely to use mobile apps [79]. In the present study, participants indicated several usability and functionality improvements, specifically the need for continually updating food product information and enhanced search/menu bar features, that will be incorporated in the future iterations of the app.

FoodFlip© app may have a public health impact beyond that of individual behaviour change. For example, FoodFlip© may allow for tracking nutritional composition of the food supply over time, if crowdsourcing was to be incorporated within the app. This data can help motivate industry to drive product reformulation towards healthier formulation. Relatedly, it may also lead industry to be more transparent about their product offerings, which can potentially help the choices of consumers with specific dietary needs (e.g. those looking for lower sodium or sugar options). Another postulated effect might be the longer-term effect of increasing nutrition knowledge or product awareness among consumers. The app may also provide insight into the perspectives of consumers, allowing for improvements in provision of informed food choice decisions.

### Strengths and limitations

Several limitations should be considered when interpreting the findings. The majority of the study participants were white, relatively highly educated with a high literacy score (measured using the Newest Vital Sign©), medium- to high-income level and the sample did not include those who did not have smartphone access suggesting that there was likely a selection bias in our study population which may decrease generalizability of the findings to the Canadian population. Additionally, the intervention was a smartphone application, using INRS that have not been implemented in Canada and it is likely there is low consumer awareness of such schemes. It is likely that findings may differ after implementation of a national front-of-pack label program. This study was not designed to assess real-time food purchases by consumers and as a result, information on the location of where a product may have been scanned (home vs. grocery store) was not collected. Additionally, the retention rate of using the smartphone app over time that would be necessary to support behaviour change requires further testing. This is especially important in the context of potential impractical replication of on-pack labels when using smartphone apps to deliver nutrition information which would require consumers to open and use the app to see front-of-pack nutrition labelling whereas, printed on-pack labels are readily apparent in the store [19]. However, on the other hand, the app may help consumers increase their nutrition knowledge and/or product awareness over longer-term, so that consumers may no longer have to consult the app as often. Research on app development recommends continuous persuasive strategies (e.g., tailored feedback), iterative development process and incorporation of behaviour change theories to increase app engagement and retention rate (long-term use of consumer nutrition mobile apps) [58, 59].

To our knowledge, this was a first study with a large sample size and randomized design to assess users’ satisfaction on a Canadian-specific smartphone app to provide consumers with easily accessible and comparable nutrition information on food and beverages. The use of a smartphone app to provide food information that consumers can potentially use in a real-world setting was innovative, however, several challenges with the use of the app were found, requiring additional modifications and testing before wide-spread implementation, particularly the need for continually updated information on product SKUs and product names. Additionally, although 95% of the participants used the app in the study, real-time usage of the app to make food comparisons may differ over time.

## Conclusion

This study is the first in Canada to explore consumers’ perceptions of different nutrition rating systems with easier-to-understand and accessible nutrition information for packaged foods delivered through a smartphone app. The results from this study suggest that that Canadian consumers may prefer a nutrient specific system such as traffic light label or ‘high in’ warning label INRS than a summary indicator system (e.g. the star rating). There was an acceptable level of self-reported user satisfaction with the FoodFlip© app with respect to provision of nutritional information that participants can use to better understand the nutrient levels. This app has the potential to support healthy dietary decision making by Canadians and can subsequently provide incentives for manufacturers to reformulate and create healthier products to achieve a more favourable rating. Future studies should investigate whether FoodFlip© with the healthfulness feature, enables real-time healthier food purchases by Canadians.

## Data Availability

The datasets used and/or analysed during the current study are not publicly available due to the wording in our informed consent forms that allowed participants to opt out of consenting to the secondary use of their data. Data from participants who consented to the secondary use of their data will be made available by the corresponding author on reasonable request.

## Abbreviations

BMI: Body Mass Index
FLIP: Food Label Information Program
INRS: Interpretative Nutrition Rating Systems
NFt: Nutrition Facts table
